# Supplementary material for: Electrical performance of a fully reconfigurable series-parallel photovoltaic module
Source: Nat Commun. 2023 Dec 8;14:8113. doi: 10.1038/s41467-023-43927-3 (PMC10703797; doi:10.1038/s41467-023-43927-3)
Supplement: Supplementary file 1 — Supplementary Information [file 41467_2023_43927_MOESM1_ESM.pdf]

Supplementary Information
Electrical performance of a fully reconfigurable series-parallel photovoltaic module

Andres Calcabrini[1], Mirco Muttillo[1], Miro Zeman[1], Patrizio Manganiello*[1], and Olindo Isabella[1]

[1]Photovoltaic Materials and Devices Group, Electrical Sustainable Energy Department, Delft University of Technology, Delft, The Netherlands. *p.manganiello@tudelft.nl

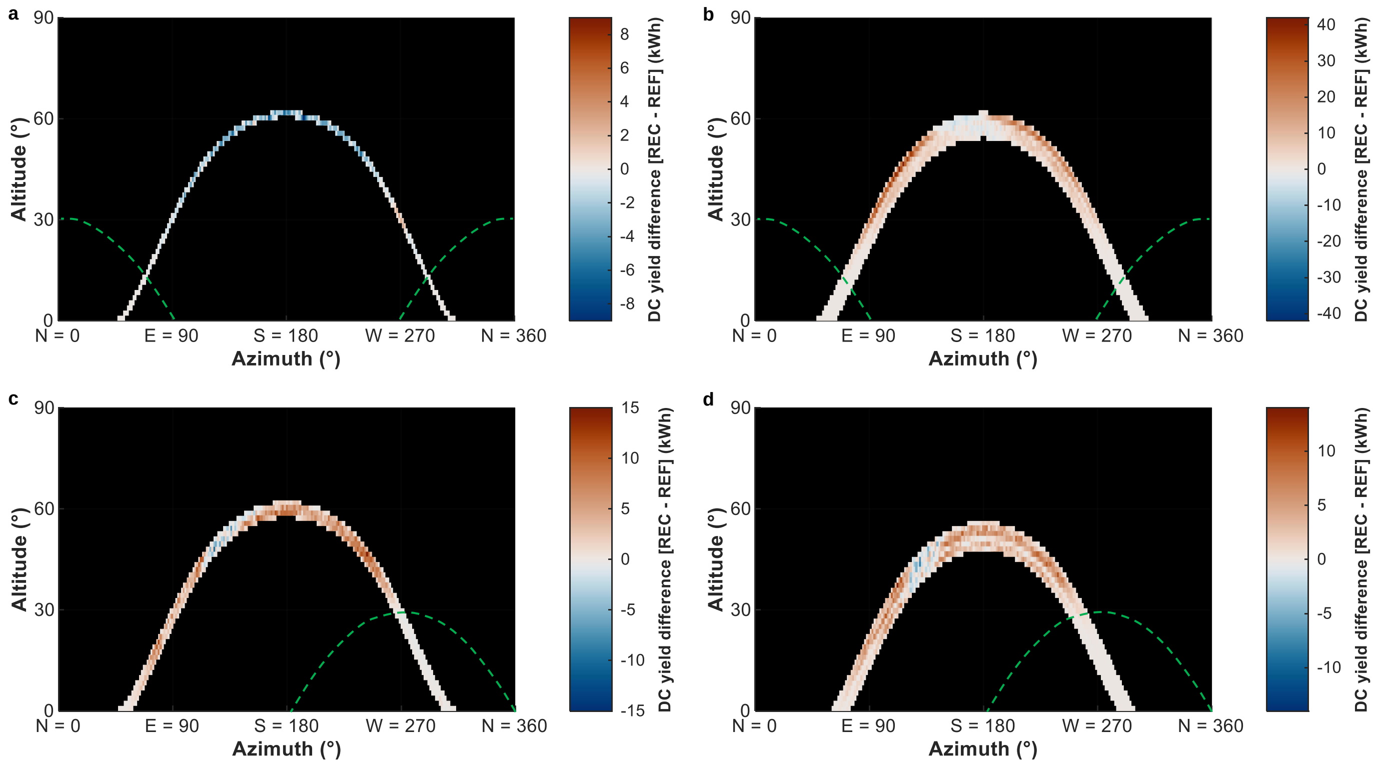

Supplementary Figure 1. Energy yield difference between the reconfigurable and reference PV module as a function of the sun position for each of the experiments: **a** Experiment 0, **b** Experiment 1, **c** Experiment 2, and **d** Experiment 3. The area below the green dashed line corresponds to the sector of the sky behind the plane of array. The start and end dates of the experiments are specified in Table 1 of the main text. Source data are provided as a Source Data file.

Supplementary Table I
BILL OF MATERIALS OF THE LAMINATED PV MODULES

Layer	Material
Front glass	Albarino T 4 mm
Front encapsulant	3M EVA9110T
Active layer	Al-BSF mono c-Si 2BB
Rear encapsulant	3M EVA9110T
Backsheet	Icosolar PPF

Supplementary Table II

TECHNICAL SPECIFICATIONS PROVIDED BY THE MANUFACTURER OF THE USED SOLAR CELLS.

Parameter	Value
Technology	Al-BSF
Area (mm^2)	153.3
Power at max. power point (W)	2.79
Voltage at max. power point (V)	0.527
Current at max. power point (A)	5.292
Open-circuit voltage (V)	0.628
Short-circuit current (A)	5.669
Temperature coefficient for Power (%/K)	-0.46
Temperature coefficient for Voltage (%/K)	-0.356
Temperature coefficient for Current (%/K)	+0.024

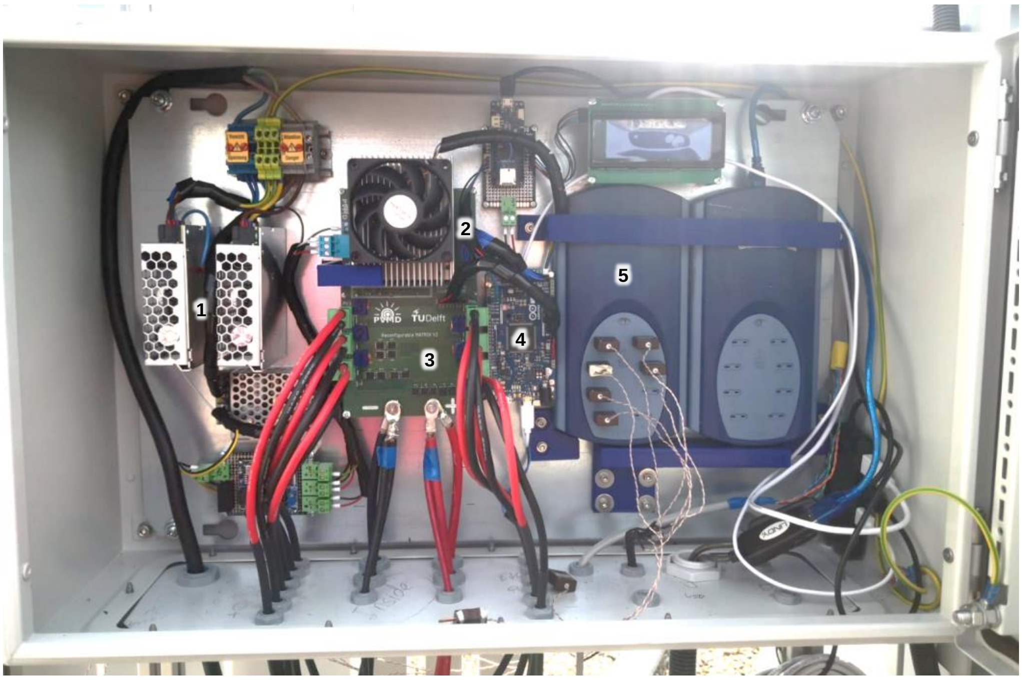

Supplementary Figure 2. Reconfiguration and sensing circuitry. (1) Power supply. The switching matrix consists of: (2) the reconfiguration board, (3) the driver circuit, and (4) the microcontroller board. (5) Temperature datalogger.

Supplementary Table III

ELECTRICAL SPECIFICATIONS OF THE EQUIPMENT USED FOR MONITORING THE ELECTRICAL PERFORMANCE OF THE RECONFIGURABLE AND REFERENCE PV MODULES. FS STANDS FOR FULL SCALE.

	Range	Resolution	Accuracy
BK8616 (REC)	0-6 A	100 µA	± (0.05% + 0.1% FS)
	0-60 A	1000 µA	± (0.05% + 0.1% FS)
	0-50 V	1 mV	± (0.05% + 0.05% FS)
	0-500 V	10 mV	± (0.05% + 0.05% FS)
PVMU (REF)	20 A	152 µA	± (2 mA + 0.1% FS)
	250 V	3.8 mV	± (0.1 mV + 0.1% FS)
