# Supplementary material for: Electrical performance of a fully reconfigurable series-parallel photovoltaic module
Source: Nat Commun. 2023 Dec 8;14:8113. doi: 10.1038/s41467-023-43927-3 (PMC10703797; doi:10.1038/s41467-023-43927-3)
Supplement: Supplementary file 3 — Reporting Summary [file 41467_2023_43927_MOESM3_ESM.pdf]

nature portfolio

Corresponding author(s): Patrizio Manganiello

Last updated by author(s): Nov 11, 2023

Reporting Summary

Nature Portfolio wishes to improve the reproducibility of the work that we publish. This form provides structure for consistency and transparency in reporting. For further information on Nature Portfolio policies, see our Editorial Policies and the Editorial Policy Checklist.

Statistics

For all statistical analyses, confirm that the following items are present in the figure legend, table legend, main text, or Methods section.

n/a	Confirmed	
☒	☐	The exact sample size (*n*) for each experimental group/condition, given as a discrete number and unit of measurement
☒	☐	A statement on whether measurements were taken from distinct samples or whether the same sample was measured repeatedly
☒	☐	The statistical test(s) used AND whether they are one- or two-sided<br>*Only common tests should be described solely by name; describe more complex techniques in the Methods section.*
☒	☐	A description of all covariates tested
☒	☐	A description of any assumptions or corrections, such as tests of normality and adjustment for multiple comparisons
☒	☐	A full description of the statistical parameters including central tendency (e.g. means) or other basic estimates (e.g. regression coefficient) AND variation (e.g. standard deviation) or associated estimates of uncertainty (e.g. confidence intervals)
☒	☐	For null hypothesis testing, the test statistic (e.g. *F*, *t*, *r*) with confidence intervals, effect sizes, degrees of freedom and *P* value noted<br>*Give P values as exact values whenever suitable.*
☒	☐	For Bayesian analysis, information on the choice of priors and Markov chain Monte Carlo settings
☒	☐	For hierarchical and complex designs, identification of the appropriate level for tests and full reporting of outcomes
☒	☐	Estimates of effect sizes (e.g. Cohen's *d*, Pearson's *r*), indicating how they were calculated

Our web collection on statistics for biologists contains articles on many of the points above.

Software and code

Policy information about availability of computer code

Data collection	The data presented in the article were collected using a commerical BK8616 electronic DC load and the LabView drivers provided by the manufacturer, and with a commercial LPVO PV monitoring unit and the LabView environment developed by the manufacturer.
Data analysis	No relevant algorithms were used to process the data presented in the plots.

For manuscripts utilizing custom algorithms or software that are central to the research but not yet described in published literature, software must be made available to editors and reviewers. We strongly encourage code deposition in a community repository (e.g. GitHub). See the Nature Portfolio guidelines for submitting code & software for further information.

Data

Policy information about availability of data

All manuscripts must include a data availability statement. This statement should provide the following information, where applicable:

- Accession codes, unique identifiers, or web links for publicly available datasets
- A description of any restrictions on data availability
- For clinical datasets or third party data, please ensure that the statement adheres to our policy

The PV module performance data generated in this study have been deposited in the 4TU.ResearchData data repository. Source data are provided with this paper.

Research involving human participants, their data, or biological material

Policy information about studies with human participants or human data. See also policy information about sex, gender (identity/presentation), and sexual orientation and race, ethnicity and racism.

Reporting on sex and gender	Not applicable.
Reporting on race, ethnicity, or other socially relevant groupings	Not applicable.
Population characteristics	Not applicable.
Recruitment	Not applicable.
Ethics oversight	Not applicable.

Note that full information on the approval of the study protocol must also be provided in the manuscript.

Field-specific reporting

Please select the one below that is the best fit for your research. If you are not sure, read the appropriate sections before making your selection.

☐ Life sciences ☐ Behavioural & social sciences ☒ Ecological, evolutionary & environmental sciences

For a reference copy of the document with all sections, see nature.com/documents/nr-reporting-summary-flat.pdf

Ecological, evolutionary & environmental sciences study design

All studies must disclose on these points even when the disclosure is negative.

Study description	The study compares the electrical performance of 2 different types of photovoltaic modules in outdoor conditions under different shading experiments over a period of approximately 4 months.
Research sample	Not applicable.
Sampling strategy	Not applicable.
Data collection	The data collection procedure was automatic and it is completely described in the article.
Timing and spatial scale	Data was collected from May 2021 to August 2021. Electrical currents and voltages of the photovoltaic modules were measured on a minutely basis. More specifically data for the four experiments discussed in the article was collected in the following intervals: [07/05/2021 - 10/06/2021], [12/06/2021 - 24/06/2021], [26/06/2021 - 22/07/2021], [03/08/2021 - 26/08/2021]. The 2-week-long gap between the last two intervals was caused by a power outage in the monitoring station that could not be quickly solved. During this gap no data was recorded at all.
Data exclusions	No data was excluded from the analysis.
Reproducibility	Four different experiments are presented in the article. No experiments failed. A reference photovoltaic module was used to establish comparison. All the necessary information about the photovoltaic modules, the measurement equipment and the measurement procedure is described in the article to ensure reproducibility of the results.
Randomization	Not applicable.
Blinding	Not applicable.

Did the study involve field work? ☒ Yes ☐ No

Field work, collection and transport

Field conditions	The most relevant environmental conditions affecting the performance of the photovoltaic modules are presented in Table 2 and Figure 8. However, it should be noted that the main conclusions of the article derive from a relative comparison between two modules that were subject to the same environmental conditions. Raw climatic data is stored in the data repository.
Location	PVMD PV monitoring station, Delft, the Netherlands. Latitude: 51.9997, Longitude: 4.3690.

Access & import/export	Not applicable.
Disturbance	Not applicable.

Reporting for specific materials, systems and methods

We require information from authors about some types of materials, experimental systems and methods used in many studies. Here, indicate whether each material, system or method listed is relevant to your study. If you are not sure if a list item applies to your research, read the appropriate section before selecting a response.

Materials & experimental systems

n/a	Involved in the study
☒	☐ Antibodies
☒	☐ Eukaryotic cell lines
☒	☐ Palaeontology and archaeology
☒	☐ Animals and other organisms
☒	☐ Clinical data
☒	☐ Dual use research of concern
☒	☐ Plants

Methods

n/a	Involved in the study
☒	☐ ChIP-seq
☒	☐ Flow cytometry
☒	☐ MRI-based neuroimaging
